# Mesoporous silica nanoparticles with an azobenzene gatekeeper as hypoxia-responsive nanocarriers for targeted doxorubicin delivery

**DOI:** 10.1007/s13346-025-01950-5

**Published:** 2025-08-29

**Authors:** Paula Rodrigo-Martínez, Mariana Barros, María Carmen Terencio, Eva Garrido, Pau Arroyo, Jose A. Sáez, Margarita Parra, Pablo Gaviña

**Affiliations:** 1 Instituto Interuniversitario de Investigación de Reconocimiento Molecular y Desarrollo Tecnológico (IDM), Universitat de València - Universitat Politècnica de València, Doctor Moliner 50, Burjassot, Valencia 46100 Spain; 2 Departamento de Farmacología, Universitat de València, Av. Vicent Andrés Estellés s/n, Burjassot, Valencia 46100 Spain; 3 Departamento de Química Orgánica, Universitat de València, C/ Doctor Moliner 50, Burjassot, Valencia 46100 Spain; 4 CIBER de Bioingeniería, Biomateriales y Nanomedicina (CIBER-BBN), Madrid, Spain

**Keywords:** Hypoxia-responsive, Mesoporous silica nanocarriers, Azoreductases, Controlled delivery, Doxorubicin, Azobenzene gatekeeper

## Abstract

Hypoxia is a key feature of solid tumors, contributing to therapeutic resistance and poor prognosis. Targeting hypoxic environments presents an opportunity to enhance drug delivery selectivity and improve treatment outcomes. Among nanocarriers, mesoporous silica nanoparticles (MSNs) have demonstrated great potential for drug delivery; however, achieving precise control over drug release remains a challenge. In this work, we develop an enzyme-responsive MSN system for targeted drug delivery in hypoxic tumors. MSNs were loaded with the chemotherapeutic drug doxorubicin (Dox) and capped with an azobenzene (Azo) gatekeeper. The bulky Azo group acts as a responsive molecular gate that remains closed under normoxic conditions but undergoes enzymatic cleavage by azoreductases, which are overexpressed in hypoxic tumor microenvironment. This cleavage triggers the uncapping of the pores, inducing the release of Dox specifically in the hypoxic region, minimizing premature drug leakage and off-target toxicity. In vitro studies with A549 cells (which overexpress azoreductase) and THP-1 cells (with low expression of azoreductases) under normoxic and hypoxic conditions, demonstrated a significant increase in Dox release and cytotoxicity in the A549 cells compared with the THP-1, which was more pronounced under hypoxia. These findings highlight the potential of enzyme-responsive MSNs as a promising strategy for selective drug delivery in hypoxic tumors.

**Graphical abstract:**

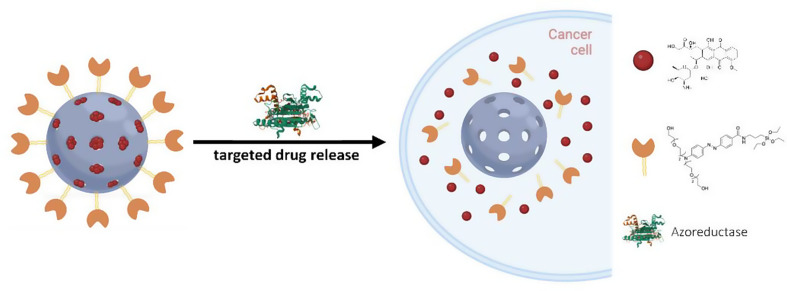

**Supplementary Information:**

The online version contains supplementary material available at 10.1007/s13346-025-01950-5.

## Introduction

Cancer, a global-threatening disease, characterized by an uncontrolled division of cells, has been ranked as the second cause of death worldwide. According to the World Health Organization, around 20 million new cases have been reported in 2022 [1]. Many efforts have been made to treat cancer, and many drugs have reached the pharmaceutical market. Still, in recent years, drug resistance has drastically increased, making long-term cancer patients’ survival more challenging [2]. Some factors cause this drug resistance, including tumor heterogeneity, tumor burden, or the hypoxia of the tumor’s microenvironment [3].

Hypoxia refers to a state where oxygen concentration is below the normal levels for a particular tissue [4]. Hypoxia is one of the hallmarks of malignant solid tumors, affecting both tumor growth and therapy effectiveness [5–7], selecting for a most malignant phenotype [8], and leading to a poor patient prognosis [9]. Most tumors larger than 1 mm^3^ contain regions of hypoxia because of the disordered blood vessel structure [10], preventing the natural diffusion of oxygen from the closest blood vessel [7, 11], and disturbing microcirculation [12]. Most malignant cancers are characterized by hypoxia, including breast cancer [13], prostate cancer [14], or lung cancer [15]. This, combined with the several side effects of chemotherapy, which include nausea, vomiting, hair loss [16], and bone marrow decrease [17] leads to the imperative need to develop new tailored delivery systems [18].

Related to the above, an increasing interest in nanotechnology has recently arisen, since it can be used as a promising tool for many purposes, such as drug delivery, medical imaging, tissue regeneration, or diagnosis [19]. Particularly, mesoporous silica nanoparticles (MSNs) have a great potential as carriers for drug delivery systems, as they feature various specific characteristics such as large specific surface areas, improved safety behavior [20], extraordinary cargo loading, tunable particle size, and easy surface functionalization [21, 22]. Their structure has a large amount of mesopores, with adjustable pore size [23], and many kinds of cargo can be loaded, from drugs to dyes. Likewise, their low toxicity compared to other metal oxides has increased the interest in these specific nanoparticles (NPs) [24]. In addition to these characteristics, the so-called enhanced permeability and retention, or EPR effect, can also facilitate the passive accumulation of MSNs in tumors, exploiting the leaky nature of their vasculature [25]. To prevent simple cargo diffusion from inside the pores to the cell environment, it’s crucial to block the pores of the MSNs by designing molecular gates (or gatekeepers) that open only in response to specific external stimuli in the hypoxic region. Many external stimuli, including changes in pH or ionic strength, enzyme activity, or light irradiation, can achieve molecular gate cleavage and cargo release [26, 27], minimizing collateral damage to healthy tissues and enhancing the precision of cancer treatments [28].

Under hypoxic conditions, an abnormal microenvironment, including a reduced pH and the over-expression of several reductases, like nitroreductase (NTR) [29], DT-diaphorase (DTD) [30], or azoreductase (AZR), is developed due to an increased reductive stress in hypoxic cells [31]. These conditions can act as a trigger for stimuli-responsive MSNs to release encapsulated drugs. The AZR-mediated reduction process is strongly determined by oxygen availability; only when low oxygen concentrations are present, like those in solid tumors, azo compounds suffer an irreversible reductive breakdown of the azo bond [32].

Recent reports have described discrete molecules including an azobenzene (Azo) cleavable moiety, for the selective delivery of anti-cancer drugs or tumor imaging [32–34]. Less are the studies that use nanoparticles combined with Azo moieties for targeting tumor hypoxia, and they are usually based on polymeric NPs [34–36], although some examples based on hybrid organic-inorganic MSNs have been reported. For instance, Lee et al. [37], and Jang et al. [38] prepared MSNs loaded with a drug and capped with a host-guest complex between Azo groups and cyclodextrin as hypoxia-responsive gatekeepers. Yan et al. reported the use of mesoporous silica nanocarriers loaded with a photosensitizer and coated with a hydrophobic azobenzene polymer and an amphiphilic copolymer, for hypoxia-responsive cargo release [39].

**Fig. 1:**
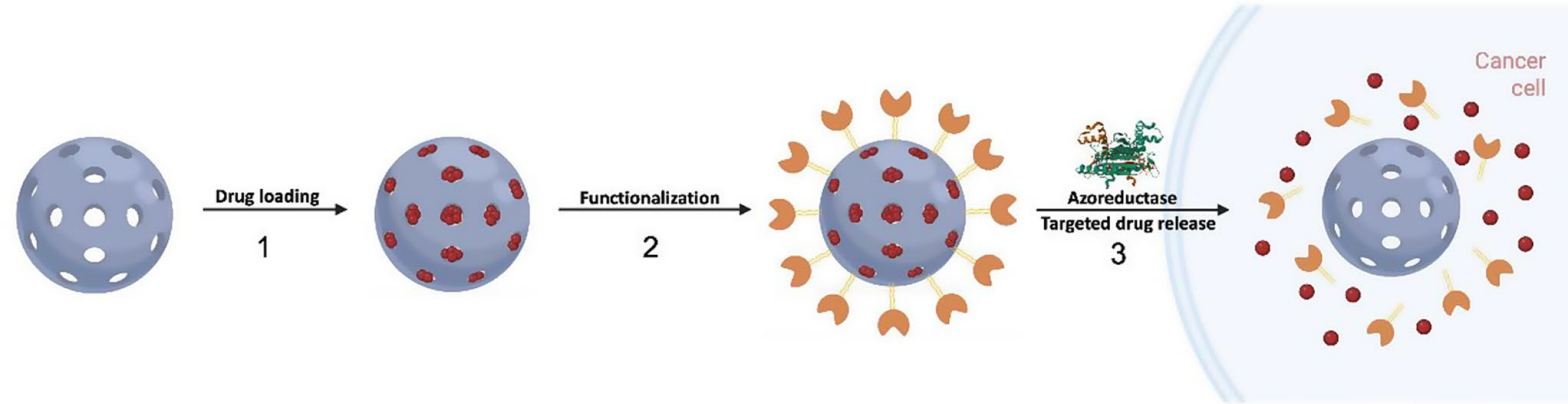
Schematic representation of the hypoxia-responsive nanocarrier assembly **S1** and the enzyme-triggered drug delivery. (1) Loading of the anticancer drug in the pores of the NPs; (2) surface functionalization with the synthesized molecular gate to achieve the final loaded nanocarrier; and (3) azoreductase-triggered Dox drug release in the hypoxic microenvironment, leading to cell death

Herein, we report the development of hypoxia-responsive gated MSNs, loaded with doxorubicin (Dox), by a facile synthesis. In our design, the gatekeeper is based on a bulky Azo derivative, directly attached to the external surface of the nanoparticles, that can be reduced by the presence of azoreductase enzymes and NAD(P)H, which are overexpressed in the solid tumor surroundings. This results in the cleavage of the Azo gate and the simultaneous opening of the pores, leading to the release of the loaded anti-cancer drug at the target site, as represented in Fig. 1.

## Materials and methods

### Materials

Aniline and cetrimonium bromide (CTAB) were purchased to Fluka Chemika. Pyridine, N’,N’-diclyclohexylcarbodiimide (DCC), N-hydroxysuccinimide (NHS), (3-aminopropyl)triethoxysilane (APTES), tetraethyl orthosilicate (TEOS), NaOH, and all standard solvents were obtained from Sigma-Aldrich. Doxorubicin hydrochloride (Dox) was acquired from Biosynth. Sodium nitrite and 4-aminobenzoic acid were purchased from Scharlau. Potassium iodide was from Panreac Quimica SA. Potassium carbonate was obtained from VWR Chemicals. 2-[2-(2-chloroethoxy)ethoxy]ethanol was purchased from TCI. ^1^H NMR and ^13^C NMR spectra were registered with Bruker Avance 300 MHz or 500 MHz spectrophotometers (Billerica, MA, USA), and have been referenced to non-deuterated solvent peak, CDCl_3,_ or DMSO-d_6_. UV-Vis spectra were registered with a Shimadzu UV-2600 spectrophotometer (Nakagyo-ku, Kyoto, Japan), using a quartz 1 cm path length cuvette. Fluorescence measurements were carried out with a Shimadzu RF-600 spectrofluorophotometer.

### Synthesis of N, N-di-(2-(2-(2-hydroxyethoxy)ethoxy)ethyl) aniline (1)

Aniline (0.93 mL, 10 mmol), KI (0.62 g, 3.73 mmol), and K_2_CO_3_ (4.84 g, 35 mmol) were dissolved in 70 mL of dimethylformamide (DMF). 2-[2-(2-chloroethoxy)ethoxy]ethanol (5 mL, 34 mmol) was added, and the suspension was stirred at 80 °C for 48 h. After completion of the reaction, the solvent was removed under reduced pressure. The crude was then redissolved in dichloromethane (DCM), washed with K_2_CO_3_ (10% w/v), brine, and water. The organic layer was dried over anhydrous MgSO_4_ and concentrated *in vacuo*. The crude product was purified by column chromatography on silica gel (AcOEt/ MeOH, 20:1) to give **1** (0.61 g, 17% yield) as a brown oil.

^1^H NMR (300 MHz, CDCl_3_) δ 7.19 (dd, *J* = 9.0, 7.1 Hz, 2 H), 6.68 (m, 3 H), 3.72–3.56 (m, 24 H) ppm. ^13^C NMR (75 MHz, CDCl_3_) δ 147.65, 129.36, 116.19, 111.74, 72.70, 70.58, 70.41, 68.63, 61.68, 50.84 ppm. HRMS: *m/z* calcd. for C_18_H_31_NO_6_ [M + H]^+^, 358.2224; found 358.2217.

### Synthesis of the intermediate azo compound 2

4-aminobenzoic acid (115.3 mg, 0.84 mmol) dissolved in 1.5 mL of water was added to a solution of HCl (37%, 0.47 mL, 15.5 mmol). A solution of NaNO_2_ (70 mg, 1 mmol) in 1.15 mL of water was added dropwise, and the reaction was stirred under an ice bath for 30 min. After this time, a solution containing **1** (300 mg, 0.84 mmol) and pyridine (284 µL, 3.53 mmol) in 10 mL of MeOH was added dropwise. The reaction mixture was left to react at room temperature for 3 h, and after this time, the solvent was removed under reduced pressure. The reaction crude was redissolved in an acetate buffer at pH 4 and washed with AcOEt. The organic layer was dried over anhydrous MgSO_4_ and concentrated *in vacuo.* Pure product **2** (350 mg, 82.4% yield) was obtained as an orange solid.

^1^H NMR (300 MHz, CDCl_3_) δ 8.13 (d, *J* = 8.9 Hz, 2 H), 7.87 (d, *J* = 8.9 Hz, 4 H), 6.78 (d, *J* = 9.0 Hz, 2 H), 3.77–3.60 (m, 24 H) ppm. ^13^C NMR (300 MHz, CDCl_3_) δ 169.88, 131.14, 129.37, 121.95, 111.55, 77.24, 72.73, 70.63, 70.45, 68.49 and 61.67 ppm.

### Synthesis of molecular gate 3

A solution of DCC (50 mg, 0.25 mmol) and NHS (28.5 mg, 0.25 mmol) in 5 mL of anhydrous THF was added to a solution of 2 (134 mg, 0.25 mmol) in 5 mL of anhydrous THF under argon atmosphere. The mixture reacted for 24 h and the excess of formed N, N’-dicyclohexylurea (DCU) was removed by centrifugation. When no more DCU was formed, APTES (60 µL, 0.25 mmol) was added to the reaction mixture and stirred overnight. After reaction completion, the solvent was removed under reduced pressure to afford molecular gate **3** (173.5 mg, 98% yield) as an orange oil.

^1^H NMR (300 MHz, DMSO-d_6_) δ 8.57 (t, J = 6.0 Hz, 1H), 7.98 (d, J = 9.0 Hz, 2 H), 7.80 (m, 4 H), 6.90 (d, J = 9.3 Hz, 2 H), 4.26 (t, J = 7.0 Hz, 2 H), 3.79–3.63 (m, 24 H), 3.54 (m, 8 H) 1.17 (m, 9 H), 0.60 (m, 2 H) ppm. HRMS: m/z calcd. for C_34_H_56_N_4_O_10_Si [M + H]^+^, 709.3844; found 709.3832.

### Synthesis of MSNs

MSNs were produced using CTAB (1.0 g, 2.74 mmol), in deionized water (480 mL) at 35 °C. NaOH (3.5 mL, 2 M) was added, and the solution was heated to 80 °C. TEOS (5 mL, 22.4 mmol) was added dropwise, and the mixture was stirred for 2 h, giving a white residue. This precipitate was centrifuged at 10,500 rpm for 10 min and washed with deionized water until neutral pH. The solid was then dried for 24 h at 70 °C. The final MSNs were obtained after calcination at 550 °C to remove the surfactant template.

## Preparation of nanocarrier S1

### Synthesis of MSN(Dox)

MSNs (20 mg) were suspended with Dox (9.9 mg, 0.8 mmol/g solid) in 700 µL of ultra-pure water. The suspension was stirred for 24 h at room temperature to load the MSN mesopores, the solid was isolated by centrifugation for 5 min at 12,000 rpm and dried under vacuum overnight, yielding **MSN(Dox)**.

### Synthesis of S1

Solid MSN(Dox) was resuspended in 700 µL of anhydrous CH_3_CN containing **3** (85 mg, 0.12 mmol, 6 mmol/g solid). The mixture was stirred for 5 h under an argon atmosphere. Then, the suspension was centrifuged at 12,000 rpm for 5 min. **S1** was washed with water and CH_3_CN to eliminate non-encapsulated cargo and unattached molecular gate. Finally, the solid was dried under vacuum.

### Characterization of MSNs and nanocarrier S1

Powder X-ray diffraction (PXRD), Fourier transform infrared (FTIR), thermogravimetric analysis (TGA), transmission electron microscopy (TEM), dynamic light scattering (DLS), and N_2_ adsorption-desorption isotherms were used to characterize both materials. The diameter and morphology of MSNs and nanocarrier **S1** were analyzed by TEM (Jeol Jem 1010), operating at 100 kV. Samples were dispersed in ultra-pure water under sonication, and then two drops were deposited onto carbon-coated copper grids. DLS of both materials was determined using a ZetaSizer Nano ZS (Malvern). FTIR spectra were registered with a Cary 630 FTIR Spectrometer (Agilent Technologies). PXRD measurements were taken on a D8 Advance diffractometer at low angles (Seifert 3000TT θ-θ) using Cu Kα radiation. Patterns (Maitenbeth, Germany) were collected in steps of 0.03° (2θ) over the angular range 0.73-10° (2θ) for 10 s per step. TGA was carried out on a TGA/SDTA 851e balance (Mettler Toledo, Columbus, OH, USA) in an oxidizing atmosphere (air, 80 mL min^− 1^) with a heating rate program between 393 and 1273 °C at 10 °C min^− 1^, followed by an isothermal heating step at 1273 °C for 30 min. N_2_ adsorption-desorption isotherms were recorded with a Tristar II Plus automated analyzer (Micromeritics, Norcross, GA, USA). The samples were degassed at 90 °C under vacuum overnight. Specific surface areas were calculated from the adsorption data within the low-pressure range using the Brunauer-Emmett-Teller (BET) model. Pore size was determined following the Barrett-Joyner-Halenda (BJH) method.

### Hydrazine-induced gate reduction

Compound **2** in EtOH solution (10 mM) was mixed with a series of hydrazine monohydrate at different concentrations (0.05, 0.10, 0.25, 0.50, 1.30, and 1.50 M), and topped up to reach 0.5 mL. After 1 h of heating at 50 °C, UV-Vis spectra of the mixture were recorded, diluting compound **2** to 3 µM.

### Controlled release assay with S1

Solid **S1** (1 mg) was suspended in PBS buffer (1 mL, pH 6.4), and this volume was divided into two aliquots of 400 µL. The aliquots were centrifuged (12,000 rpm, 4 min), and the fluorescence of the supernatant at 595 nm was measured (λ_exc_ = 470 nm) for the obtention of the “zero point”. Then, 2 µL of hydrazine monohydrate was added to the measurement aliquot, and the samples were filled to 500 µL with more PBS buffer. Both samples were then shaken at 37 °C and 750 rpm. After a pre-determined time, aliquots were centrifuged at 12,000 rpm for 4 min. The fluorescence of the released Dox at 595 nm (λ_exc_ = 470 nm) was measured in the supernatant (120 µL). The 120 µL aliquot was then returned to the initial suspension.

### Cell culture conditions

Human lung cancer A549 cells and human monocyte THP-1 cells were grown in Dulbecco’s Modified Eagle Medium (DMEM) (Gibco™, Paisley, UK) with 10% fetal bovine serum (FBS) and supplemented with penicillin 100 U/mL and streptomycin 100 µg/mL (Gibco™, Paisley, UK). A day before the experiment, the cells were harvested by utilizing 0.25% trypsin-EDTA and planted at a density necessary by individual studies. This allowed the cells to reacclimate.

THP-1 human monocytic cells were cultured in Roswell Park Memorial Institute (RPMI) medium (Gibco™, Paisley, UK) supplemented with 10% FBS and the same penicillin/streptomycin mixture. Differentiation of THP-1 to macrophages was performed by incubating them with 12-*O*-tetradecanoyl phorbol 13-acetate (TPA) 10 nM for three days.

### Cytotoxicity tests

3-(4,5-dimethylthiazol-2-yl)-2,5-diphenyl tetrazole bromide (MTT) method was used to assess the cell toxicity of the blank nanoparticles and loaded-DOX nanocarrier. A 96-well plate (Sarstedt^®^, Nümbrecht, Germany) was seeded with A549 cells (50,000 cells per well) or THP-derived macrophage cells (200,000 cells per well) in DMEM with 10% FBS (final volume 200 µL/well). After 24 h– incubation to allow cell attachment, medium was replaced by DMEM (1% FBS) supplemented with various quantities of empty gated MSNs (**S2**, unloaded nanocarrier) and DOX-loaded nanocarrier **S1** (1–100 µg/mL), and the cells were further incubated during 24 h. Next, the cells were washed with PBS to remove any residual dyes physically absorbed on the cell surface, and 100 µL of MTT (Sigma-Aldrich, St. Louis, Missouri, USA) dissolved in culture medium (500 µg/mL) was added. After incubation for 1 h at 37 °C, the medium was removed, and 100 µL DMSO was added to each well to dissolve the formazan crystals. The absorbance was measured at 531 nm using a spectrophotometer VICTOR3™ V1420 (PerkinElmer, Finland).

In another set of experiments, A549 cells were seeded and treated as above, but incubated with **S1**,** S2** and free-Dox under hypoxic conditions (2% O_2_) in a hypoxia chamber BioSpherix Xvivo System. After 24 h-incubation, MTT assays were also performed.

The results are presented as the mean ± standard deviation (SD) of the values. The statistical treatment employed was the one-way ANOVA method, followed by Dunnet’s multiple comparison test. Data analysis was performed using the GraphPad Prism 5 program (GraphPad Software, La Jolla, CA, USA).

### Confocal fluorescence microscopic images

A549 cells were seeded at a density of 50,000 cells per well in a cell culture Chamber Slide™, in DMEM with 10% FBS, and incubated for 24 h for cell attachment. The medium was then replaced with DMEM containing 1% FBS, and the cells were incubated with **S1** at concentrations of 25 and 50 µg/mL for 4 and 24 h. To visualize the mitochondria and enhance cytosolic definition,4 µL of MitoTracker™ Green Dye for Mitochondria Labelling (Molecular Probes™ Invitrogen, Paisley, UK) (5·10^4^ nM) was added to the medium for the last 30 min. Finally, the cells were washed three times with PBS to remove unbound nanoparticles, and to visualize the nuclei, a drop of ProLong™ Gold Antifade Mountant mounting fluid with 4’,6-diamidino-2-phenylindole (DAPI) (Molecular Probes™ Invitrogen, Paisley, UK) was added. The slides were observed in a Confocal Multiphoton FV1000MPE Microscope with a 40X objective and Alexa Fluor 488, Texas Red, and DAPI channels. In a parallel experiment, the cells were incubated during 24 h with **S1** (25 and 50 µg/mL) under hypoxic conditions (2% O_2_) and confocal imaging was observed with ProLong™ Gold Antifade Mountant mounting fluid DAPI, in an LSM980 Microscope with a 40X objective and Texas Red and DAPI channels. Quantification and data treatment were carried out using the ImageJ2 (version 2.16.0/1.54p) software.

## Results and discussion

### Design and synthesis of the azoreductase-responsive gated nanocarrier S1

**Scheme 1:**
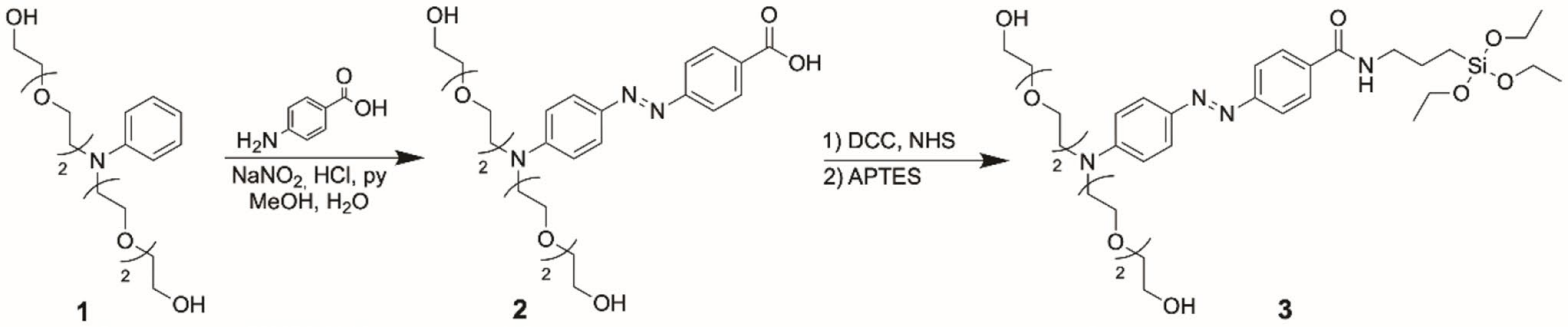
Synthetic route for molecular gate **3**

As mentioned above, our goal was the design and synthesis of a nanodevice for drug release at the desired site, based on the overexpression of AZRs in the hypoxic microenvironment. For this purpose, molecular gate **3** (see Scheme 1) incorporating an Azo moiety as the AZR-responsive linker, two oligo(ethylene glycol) chains as bulky and solubilizing groups, and a trialcoxysilane as reactive group to form Si-O-Si bonds with the silanol groups on the silica surface, was designed, to be subsequently anchored to the synthesized MSNs. On the one hand, gatekeeper **3** would block the nanoparticle’s pores, avoiding the release of the cargo; on the other hand, the Azo moiety can be reductively cleaved in the hypoxic microenvironment, releasing the drug. In detail, molecular gate **3** was obtained in a three-step synthesis (Scheme 1), starting with the dialkylation of aniline with an excess of 2-[2-(2-chloroethoxy)ethoxy]ethanol, to obtain product **1**. Afterward, **1** underwent a diazotization-coupling reaction [32] with 4-aminobenzoic acid in an acidic solution to yield product **2**. Finally, APTES was made to react with product **2** in the presence of DCC and NHS, in an inert atmosphere to give the carbamate bridge, and the complete molecular gate **3**. All these compounds were confirmed by nuclear magnetic resonance and mass spectrometry experiments.

The starting MSNs were synthesized, by a sol-gel condensation of TEOS in the presence of sodium hydroxide and CTAB as a micellar template, following conditions published elsewhere [22]. Once MSNs were calcinated at 550 °C, the pores were loaded with the chemotherapeutic drug Dox, in aqueous solution. After cargo loading, the synthesized molecular gate **3** was anchored onto the external surface of the MSNs, through the condensation reaction between the silanol groups of the MSN and the triethoxysilyl groups of the molecular gate, giving the solid **S1**, in which the release of the encapsulated cargo is avoided because of pore blockage.

### Characterization of the prepared nanomaterials

**Fig. 2:**
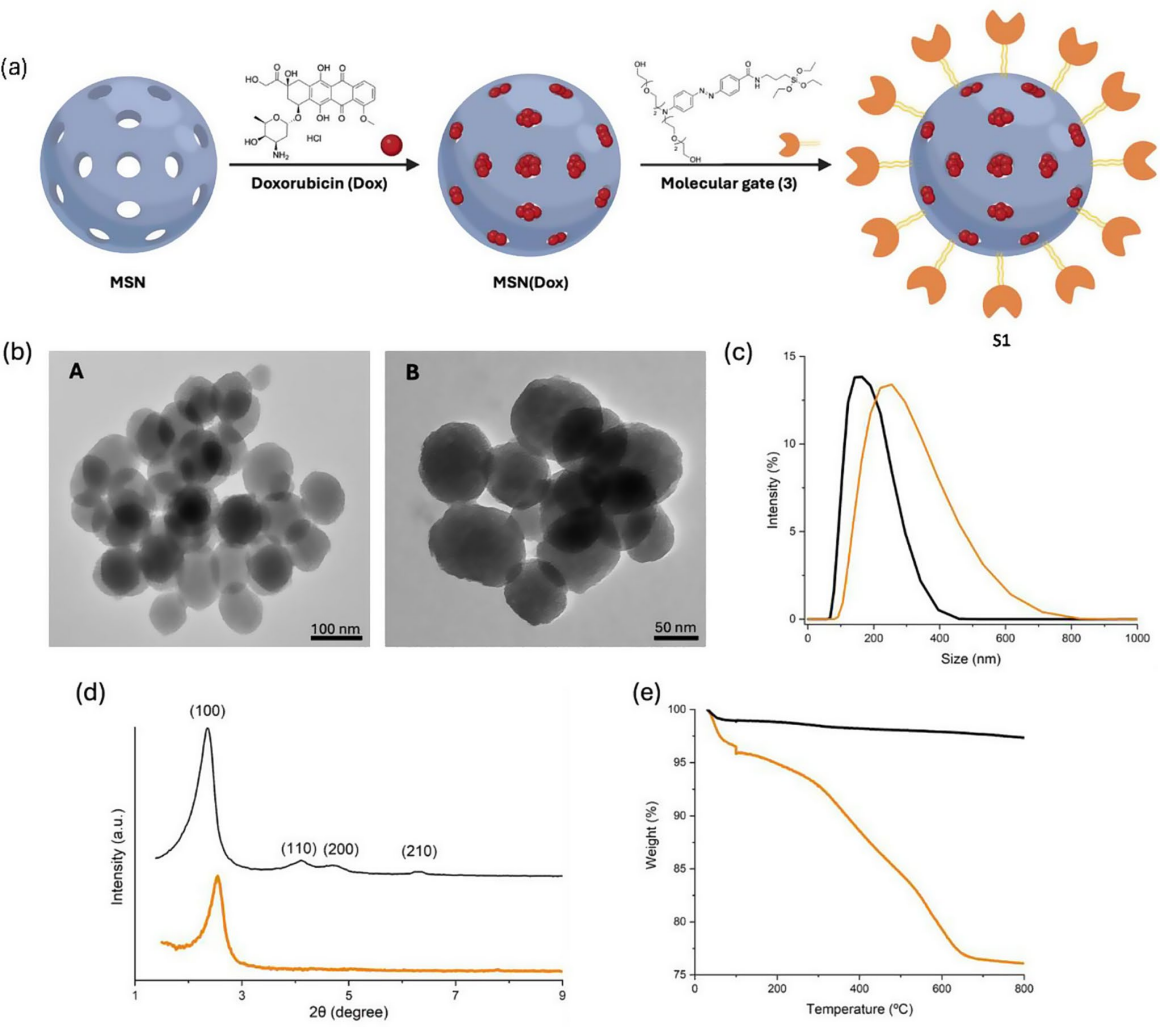
Physicochemical characterization of MSNs and hypoxia-responsive nanocarrier **S1**. (**a**) Schematic synthetic procedure for **S1**; (**b**) TEM images of (A) calcinated MSNs and (B) **S1**; (**c**) Hydrodynamic diameter distribution determined by DLS for calcinated MSN (black line) and **S1** (orange line); (**d**) Powder X-ray diffraction patterns of the MSN (black line) and **S1** (orange line); (**e**) Thermogravimetric analysis for MSN (black line) and **S1** (orange line)

To characterize the synthesized nanomaterials, several techniques, such as PXRD, TEM, DLS, N_2_ adsorption-desorption isotherms, FTIR, and TGA analyses, were used. The particle size and shape of the synthesized nanostructure were studied by TEM (Fig. 2b), showing a high internal porous ordering at the nanoscale for the MSNs observed as black and white stripes; both MSN and **S1** show a spherical morphology (See Figure S4). An average diameter of 97.81 ± 7.0 nm (*n* = 30 particles) was calculated, similar to our previous studies [22] confirming that the siliceous matrix remains unchanged when loading and capping the MSNs. The size of the solid **S1** assembly was assessed through DLS measurements (Fig. 2c). In this respect, 162.7 ± 3 nm was obtained as a starting hydrodynamic diameter for the MSNs, and after cargo loading and functionalization, the hydrodynamic diameter increased to 242.1 ± 5 nm. This increase ratifies the expected presence of the anchored molecular gate on the MSN surface. In the XRD diffractograms of MSN-based materials (Fig. 2d), the unmodified MSN shows an intense Bragg peak, corresponding to (100) plane, and three low-intensity diffraction Bragg peaks, corresponding to (110), (200), and (210) planes, respectively. After functionalization, a decrease in the intensity of the (100) peak can be observed, as well as a broadening of the (110) and (200) peaks, due to the loss of contrast by filling the pores with the cargo. The FTIR spectrum of **S1** (Figure S6) shows the appearance of signals corresponding to aromatic = C-H bonds (> 3000 cm^− 1^), C = C bonds and N = N bonds (ca. 1600 cm^− 1^) and the characteristic peak for Si-O-Si at ca. 1050 cm^− 1^. Furthermore, the zeta potential values for calcined MSNs and **S1** are − 21.6 *±* 0.1 mV and + 31.3 *±* 0.4 mV, respectively. The negative value for the zeta potential of the calcined MSNs is attributed to the presence of silanolate anions on the surface of the nanoparticle. However, after functionalization with molecular gate **3**, zeta potential becomes positive in **S1**. From the N_2_ adsorption-desorption isotherms, a BET-specific surface area of 1060 m^2^ g^− 1^ for the calcinated MSNs was measured, and an average pore size of ca. 2.85 nm (See Figure S5).

The cargo loading was determined from delivery studies and thermogravimetric analyses (Fig. 2e). In this respect, solid **S1** contains 62.5 mg of Dox per gram of solid, which corresponds to a drug loading content (DLC) of 6.25% w/w. We found in the literature that for similar gated MSNs, Dox DLC values range from approximately 5–15% w/w, depending on the functionalization and loading methods used [40–42]. Thus, our DLC falls within the typical range for similar systems. This level of drug loading is considered suitable for effective drug delivery, as it balances therapeutic efficacy with reduced cytotoxicity and supports system stability and controlled release.

### Reduction of molecular gate 3 under mimicked hypoxic environment

**Fig. 3:**
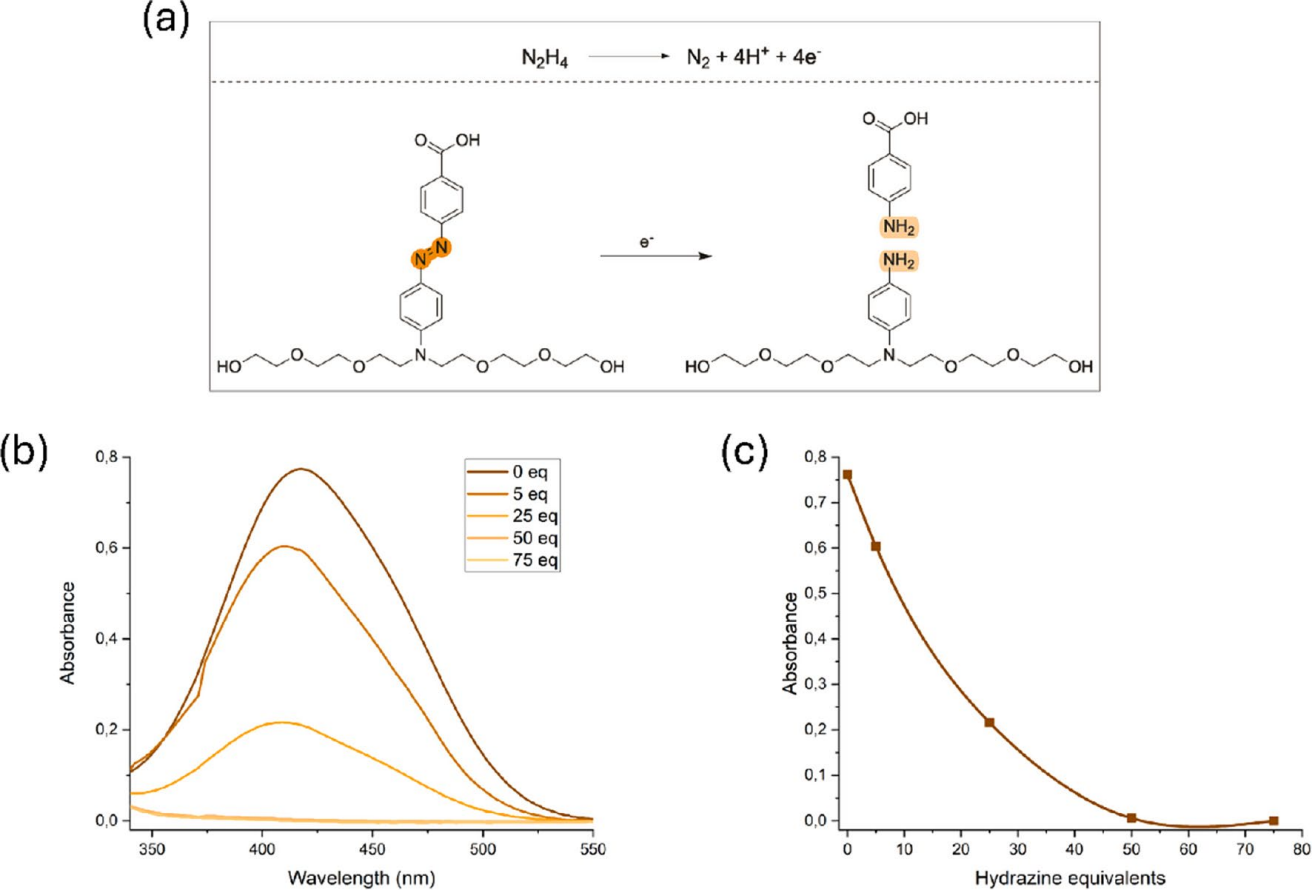
(**a**) Proposed mechanism of hydrazine-induced reduction of molecule **2**; (**b**) UV-vis absorbance spectra changes of molecule **2** (10 mM in EtOH) after treatment with increasing concentrations of hydrazine monohydrate (0–75 equiv.); (**c**) Changes in absorbance intensity at 417 nm as a function of hydrazine concentration

The effective reduction of the Azo moiety requires the presence of AZR and nicotinamide adenine dinucleotide phosphate (NADPH) as cofactor, both present in hypoxia tumor environments. For in vitro experiments, we selected hydrazine monohydrate as the chemical reducing agent to test the successful reduction of Azo. The expected mechanism for the reduction of **2** is shown in Fig. 3a. In the presence of hydrazine, a decrease in the characteristic UV-Vis band of Azo moiety is observed in Fig. 3b. The extent of this decrease is proportional to the concentration of the reducing agent, achieving a total reduction of the Azo moiety at 50 equivalents of hydrazine, as can be seen in Fig. 3c. This reduction can also be observed with the naked eye since the Azo is bright orange, and color loss is observed by adding hydrazine (See Figure S7).

### Drug release of Dox from S1 under reducing conditions

**Fig. 4:**
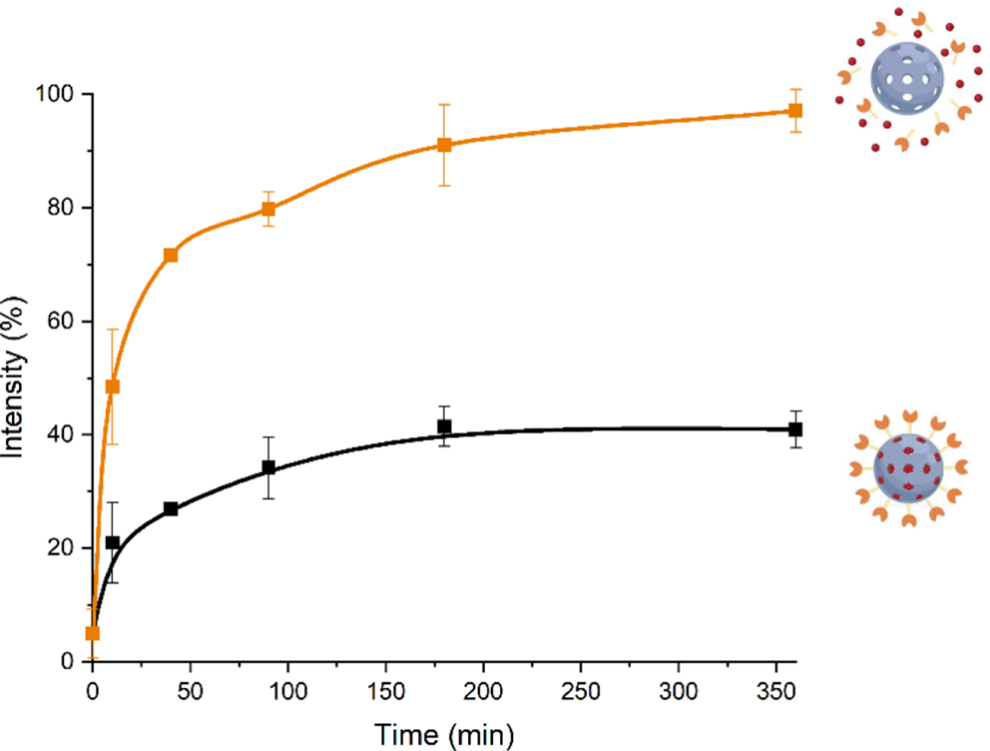
Delivery profiles of Dox from the solid **S1** after 6 h in PBS at pH 6.4 in the absence (black line) and in the presence (orange line) of hydrazine monohydrate (1 µL/mL)

Azo moiety in the molecular gate **3** can be rapidly reduced by the AZRs present in hypoxic tumor environments allowing the release of the Dox contained in MSN mesopores. To examine the Dox release profile of **S1** under simulated hypoxic conditions, we used hydrazine monohydrate in PBS solution (500 µL, at pH 6.4). To confirm our strategy, cargo delivery from **S1** in the absence of reducing agent (blank) was also studied in PBS solution (pH 6.4) comparing the obtained results (see Materials and methods section). As can be observed in Fig. 4, in the absence of hydrazine, a low cargo delivery can be seen. In contrast, in the presence of hydrazine, a remarkable release of Dox is found after 2 h. This cargo release is due to the reduction of Azo into two amine groups, leaving enough space for the Dox to come out of the mesopores.

### Cytotoxic activity in azoreductase overexpressed cancer cells compared to monocyte-macrophage cells

After verifying that nanocarriers **S1** preferentially release their cargo in a reducing microenvironment, we evaluated the efficacy of the nanodevice for the targeted release of anti-cancer drugs to azoreductase-overexpressing cells. In a first stage, azoreductase overexpressed cells, A549 (human nonsmall cell lung carcinoma cell line), and THP-1 cells (human monocytic cell-line with low expression of AZR) were employed for cell viability assay under normoxic conditions. THP-1 cell line presents an additional interest to study since free Dox significantly affects monocyte and macrophage survival, function, and differentiation in cancer patients, producing some side effects like immune system suppression [43].

**Fig. 5:**
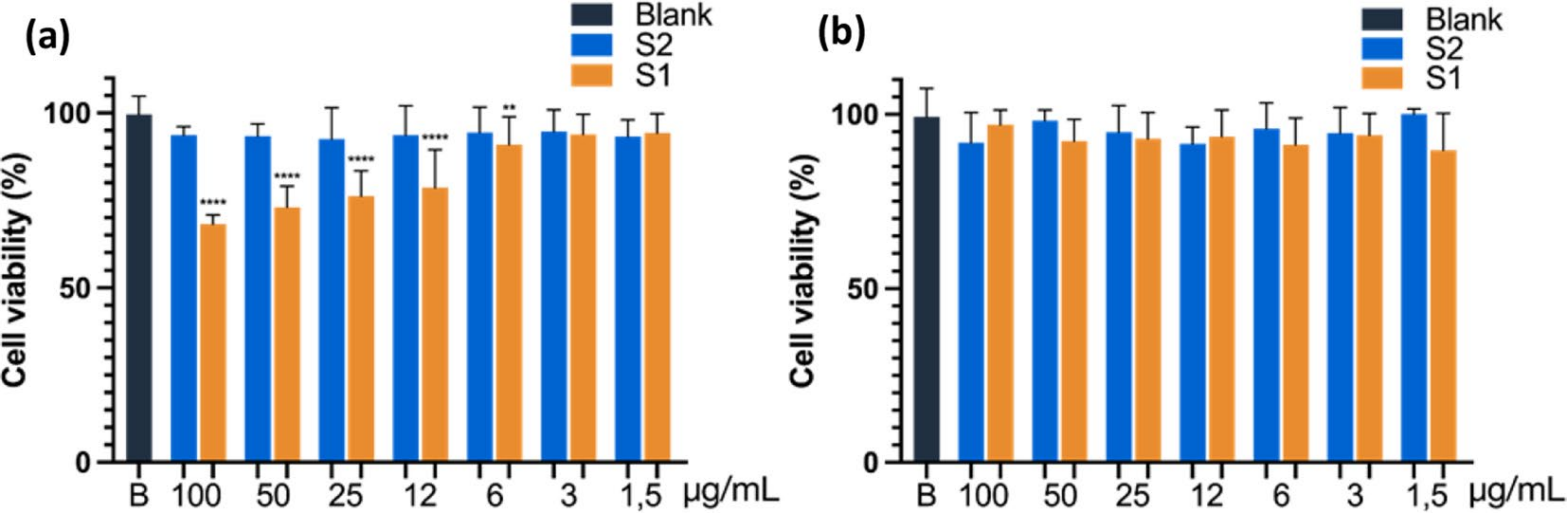
Cell viability of unloaded (blue, **S2**), and Dox-loaded (orange, **S1**) nanoparticles in (**a**) A549 lung cancer cells and (**b**) THP-1 human monocyte cells. Results are expressed as mean ± SD (*n* = 15). *p < 0.05, ****p < 0.0001 versus control untreated cells (Blank). One-way ANOVA followed by Dunnet’s test

First, it is of the greatest importance to test the cell viability of the gated nanoparticle itself. For that, the cells were individually treated with variable concentrations of empty nanoassemblies **S2** (MSNs capped with the Azo gatekeeper, but without cargo) and incubated for 24 h. The cell viability was measured using the MTT assay. Interestingly, the nanoparticle itself exhibits high cell viability in both cell lines (Fig. 5, blue columns). This implies that the nanoparticle has a high biocompatibility and is a potential material for chemotherapeutic applications in cancer. Next, cytotoxicity of the Dox-loaded nanoassemblies **S1** toward A549 cancer cells and THP-1 cells, was tested by incubation for 24 h. As expected, results revealed a significant and concentration-dependent cell death for azoreductase overexpressed cancer cells (A549, Fig. 5a) even at low concentrations (3 µg/mL), while there is no significant cell death for THP-1 cells (Fig. 5b). This confirms that the azoreductase enzyme plays a major role in reducing the azo bond in the molecular gate, leading to the release of Dox molecules to the cytosol of the cancer cells while showing a benign nature toward normal cells, making the system very interesting and promising in the field of targeted therapeutic applications. Besides, the lack of **S1** cytotoxicity in THP-1 cells, even at the highest concentration, would suggest the absence of an unspecific passive doxorubicin release from the loaded nanoparticles.

### Confocal microscopy imaging

The A549 cells were used to evaluate the cellular uptake of the Dox-loaded NPs **S1** by confocal laser scanning microscope. Two concentrations (25 and 50 µg/mL) and two incubation times (4 and 24 h) were used to perform a comparative analysis. As shown in Figure S8, a strong and concentration-dependent red fluorescence was observed after 4 h-incubation with the NPs, indicating their internalization and accumulation into the cells, mainly near the mitochondria and around the nucleus (merged image). After 24 h of incubation, the concentration-dependent red fluorescence was accompanied by cellular death with nuclear damage and mitochondrial fragmentation, which can be observed easily with the 50 µg/mL treatment (Figure S9). These results were also confirmed by quantitative analysis of images, which showed a significant increase of Dox fluorescence in the 50 µg/mL treatment compared to the 25 µg/mL treatment; the fluorescence intensity is maintained in the time (See Figure S10).

Despite the mitochondrial and nuclear damage observed in the confocal images, the uptake of doxorubicin into the nucleus was not clearly observed under our experimental normoxic conditions. Thus, we decided to perform new experiments under hypoxia conditions (2% O_2_) to enhance azoreductase overexpression in A549 cells and consequently increase the gate opening of nanoassemblies.

### Cytotoxicity studies in A549 cancer cells under hypoxia conditions

**Fig. 6:**
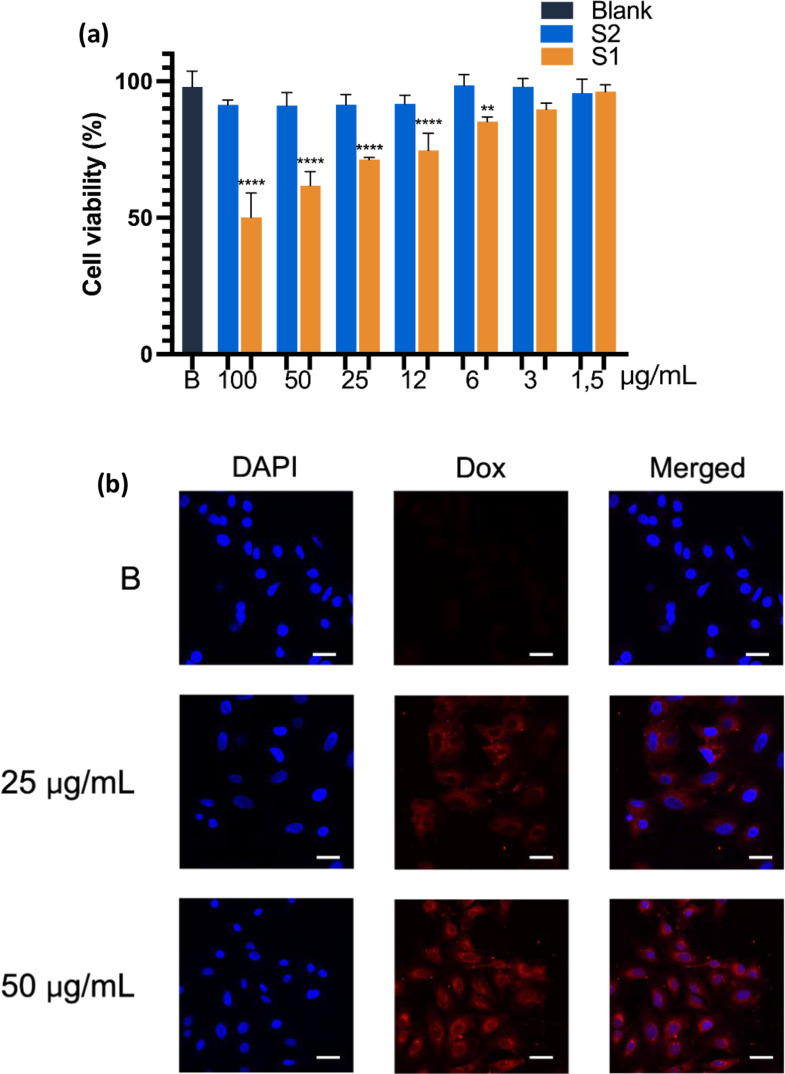
(**a**) Cytotoxicity of unloaded blank (blue **S2**) and Dox-loaded (orange, **S1**) in A549 lung cancer cells after 24 h-incubation under hypoxic conditions (2% O_2_). Results are expressed as mean ± SD (*n* = 3). **p < 0.01, ****p < 0.0001 versus Blank untreated cells (B). One-way ANOVA followed by Dunnet’s test. (**b**) Confocal microscope images of A549 cells after 24-incubation with S1 (25 and 50 µg/mL). B: untreated cells. Cell nuclei were stained with DAPI. Pink stain: colocalization of Dox within the nuclei. Representative image from three independent experiments. Untreated Scale bar 20 μm

In this set of experiments, A549 cells were incubated with **S1** (Dox-loaded) and **S2** (unloaded) during 24 h under hypoxia conditions (2% O_2_). As shown in Fig. 6a, results of MTT assay confirmed the absence of cytotoxicity of empty nanoassemblies. In contrast, **S1** produced a clear increase in cytotoxicity compared to normoxic conditions, with an IC_50_ value near 100 µg/ml.

Since the loading of Dox into the NPs was approximately 6%, a new study was performed by incubating the cells during 24 h with free doxorubicin at 1.5, 3 and 6 µg/ml (concentrations equivalent to 25, 50 and 100 µg/ml of Dox-loaded nanoassemblies). Results showed a concentration-dependent cytotoxic effect for free Dox in a similar range to Dox-loaded nanoparticles **S1**, although free doxorubicin only reduced cell viability to 35% at the highest concentration tested (which is equivalent to 100 µg/ml of Dox entrapped in **S1**) (Figure S11).

A possible explanation for the somewhat lower activity of free Dox respect to DOX-loaded nanoparticles is that nanoparticles are more readily internalized by an endocytosis mechanism compared to a passive diffusion mechanism of Dox into cells [44].

Figure6 shows confocal images of cells treated with **S1** (25 and 50 µg/ml) under hypoxia conditions. After 24 h of incubation, we observed a concentration-dependent red fluorescence into the cells, which was confirmed by quantitative analysis of images (Figure S12). In addition, a clear increase of nuclear uptake of Dox into the nucleus was detected, mainly at 50 µg/mL. These results confirm that hypoxia conditions in A549 cells enhance the release of Dox from the loaded NPs due to the increase of azoreductase overexpression and the consequent gate opening.

## Conclusions

In summary, a hypoxia-responsive mesoporous silica nanocarrier, loaded with the chemotherapeutic drug Dox, and capped with an Azo-based gatekeeper incorporating two oligo(ethylene glycol) chains has been synthesized and characterized by TEM, PXRD, DLS, N_2_ adsorption-desorption isotherms, FTIR, and TGA analysis. UV-Vis and fluorescence experiments demonstrate that the molecular gate avoids cargo release due to the capping of the pores, but under reductive conditions undergoes reduction of the azo bond, triggering the delivery of the cargo. In vitro cytotoxicity and confocal microscopy studies of the doxorubicin-loaded nanocarrier **S1** with A549 cells, which overexpress azoreductases, and THP-1 cell-line, with low expression of azoreductases, revealed selective cytotoxicity toward azoreductase overexpressed cancer cells. In addition, hypoxic conditions enhance cytotoxicity and nuclear damage in A549 tumoral cells by the increase of gate-opening and doxorubicin release from **S1**. For 50 µg/mL of **S1** in normoxic conditions, cell viability after 24 h was found to be 73% whereas in hypoxic conditions, cell viability decreased to 60%. The results shown in this research demonstrate that azobenzene-containing gatekeepers constitute an interesting motif for developing enzyme-responsive gated nanostructures. Although further validation in vivo is required, these preliminary in vitro results highlight the potential of enzyme-responsive gated MSNs as a promising strategy for selective drug delivery in hypoxic tumors, offering improved therapeutic efficacy and reduced side effects compared to conventional chemotherapy.

## Supplementary Information

Below is the link to the electronic supplementary material.

Supplementary Material 1

## Data Availability

All data are included in the manuscript or in the Supplementary Information.
