# Supplementary material for: Mesoporous silica nanoparticles with an azobenzene gatekeeper as hypoxia-responsive nanocarriers for targeted doxorubicin delivery
Source: Drug Deliv Transl Res. 2025 Aug 29;16(5):1423–34. doi: 10.1007/s13346-025-01950-5 (PMC13038766; doi:10.1007/s13346-025-01950-5)
Supplement: Supplementary file 1 — Supplementary Material 1 [file 13346_2025_1950_MOESM1_ESM.docx]

Supplementary Material

Mesoporous silica nanoparticles with an azobenzene gatekeeper as hypoxia-responsive nanocarriers for targeted doxorubicin delivery

Paula Rodrigo-Martínez,^1^ Mariana Barros,^1^ Maria Carmen Terencio,^1,2^ Eva Garrido,^1,4^ Pau Arroyo,^1,3^ Jose A. Sáez,^1,3^ Margarita Parra^1,3,4^ and Pablo Gaviña,^1,3,4,^*

^1^ Instituto Interuniversitario de Investigación de Reconocimiento Molecular y Desarrollo Tecnológico (IDM), Universitat Politècnica de València, Universitat de València, C/ Doctor Moliner 50, Burjassot, 46100, Burjassot, Valencia, Spain.

^2^ Departamento de Farmacología, Universitat de València, Av. Vicent Andrés Estellés s/n, Burjassot, 46100, Valencia, Spain.

^3^ Departamento de Química Orgánica, Universitat de València, C/ Doctor Moliner 50, Burjassot, 46100, Burjassot, Valencia, Spain.

^4^ CIBER de Bioingeniería, Biomateriales y Nanomedicina (CIBER-BBN), Madrid, Spain

**Contents**

[**Figure S1.** (a) ^1^H NMR, (b) ^13^C NMR, and (c) mass spectroscopy of Compound **1**. 2](#_Toc203992802)

[**Figure S2**. (a) ^1^H NMR, and (b) ^13^C NMR of Compound **2**. 3](#_Toc203992803)

[**Figure S3.** (a) ^1^H NMR, and (b) mass spectroscopy of Compound **3**. 4](#_Toc203992804)

[**Figure S4.** TEM images of (a) as made MCM-41 and (b) S1 nanoparticles, at three different distances. 5](#_Toc203992805)

[**Figure S5.** N_2_ adsorption-desorption isotherms for calcinated MSN. 5](#_Toc203992806)

[**Figure S6.** FTIR spectra of MSN (black) and S1 (brown). 6](#_Toc203992807)

[**Figure S7.** Naked-eye changes in the reduction of Compound 2 (10 mM in EtOH) with the treatment of increasing concentrations of hydrazine monohydrate (from left to right, 0-75 equiv.). 6](#_Toc203992808)

[**Figure S8.** Confocal microscope images of A549 untreated cells (B) and A549 cells after incubation with different concentrations of **S1** for 4 and 24 h respectively. Cell nuclei were stained with DAPI and mitochondria with MitoTracker Green. Representative image from three independent experiments. Scale bar 20 μm. 7](#_Toc203992809)

[**Figure S9.** 2.2X zoom of the 24 h 50 μg/mL treatment confocal image in Figure S8. Scale bar 20 μm. 7](#_Toc203992810)

[**Figure S10.** Quantitative analysis of Dox fluorescence intensity per field after confocal imaging in A549 cells incubated for 4 and 24h with S1 (25 – 50 μg/mL). Results are expressed as mean ± SD (n = 10).****p<0.0001 Student Test. 8](#_Toc203992811)

[**Figure S11.** Cytotoxicity of free Dox in A549 lung cancer cells after 24h-incubation under hypoxic conditions (2% O_2_). Results are expressed as mean ± SD **(n=3).** ****p< 0.0001 versus Blank untreated cells (B). One-way ANOVA followed by Dunnet’s test. 8](#_Toc203992812)

[**Figure S12.** Quantitative analysis of Dox fluorescence intensity after confocal imaging in A549 cells incubated for 24h under hypoxia conditions with S1 (25 – 50 μg/mL). Results are expressed as mean ± SD (n = 10).****p<0.0001 Student Test. 8](#_Toc203992813)

(a)

(b)

(c)

**Figure S1.** (a) ^1^H NMR, (b) ^13^C NMR, and (c) mass spectroscopy of Compound **1**.

(a)

(b)

**Figure S2**. (a) ^1^H NMR, and (b) ^13^C NMR of Compound **2**.

(a)


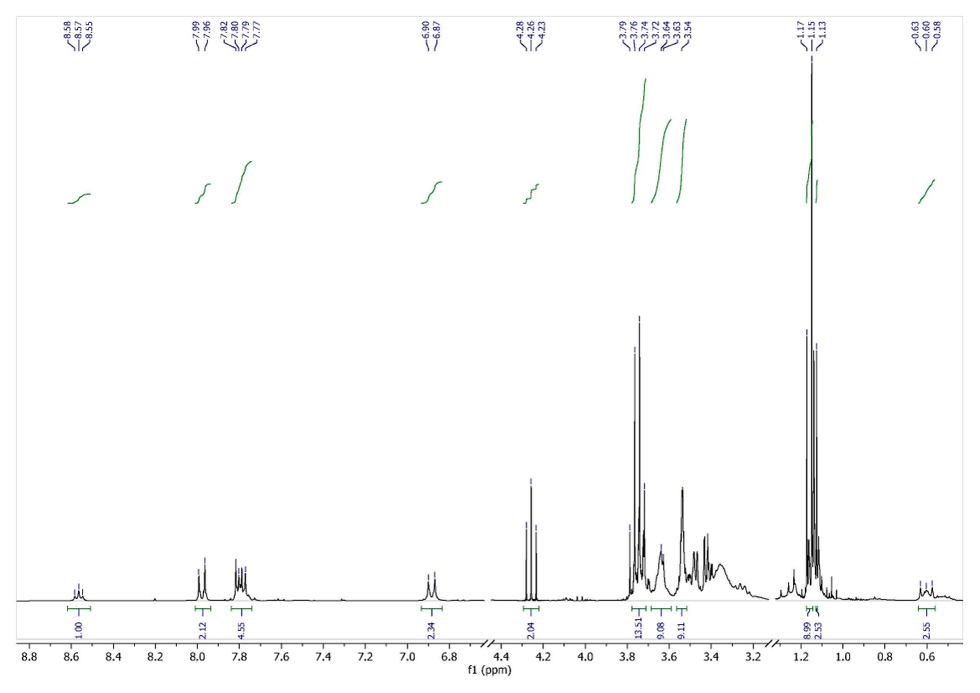


(b)

**Figure S3.** (a) ^1^H NMR, and (b) mass spectroscopy of Compound **3**.

(a)


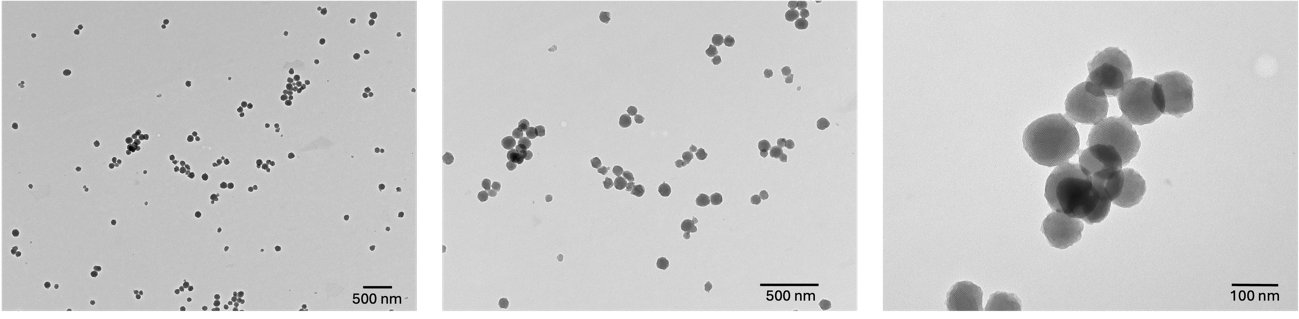


(b)


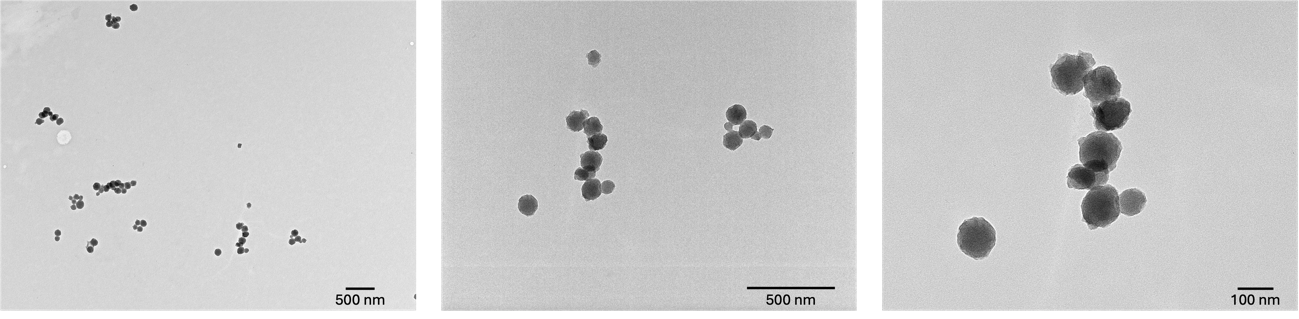


**Figure S4.** TEM images of (a) as made MCM-41 and (b) S1 nanoparticles, at three different distances.





**Figure S5.** N_2_ adsorption-desorption isotherms for calcinated MSN.


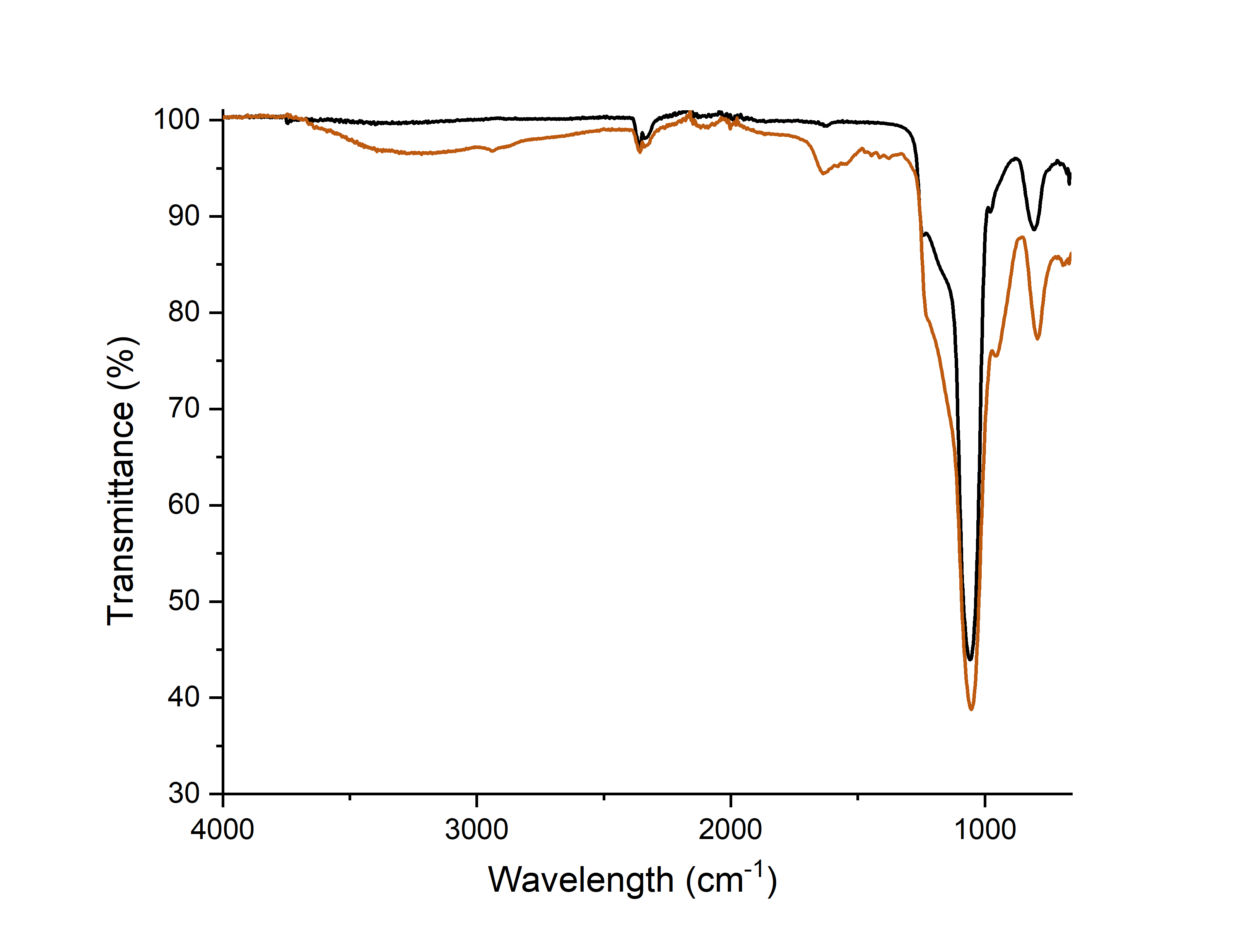


**Figure S6.** FTIR spectra of MSN (black) and S1 (brown).


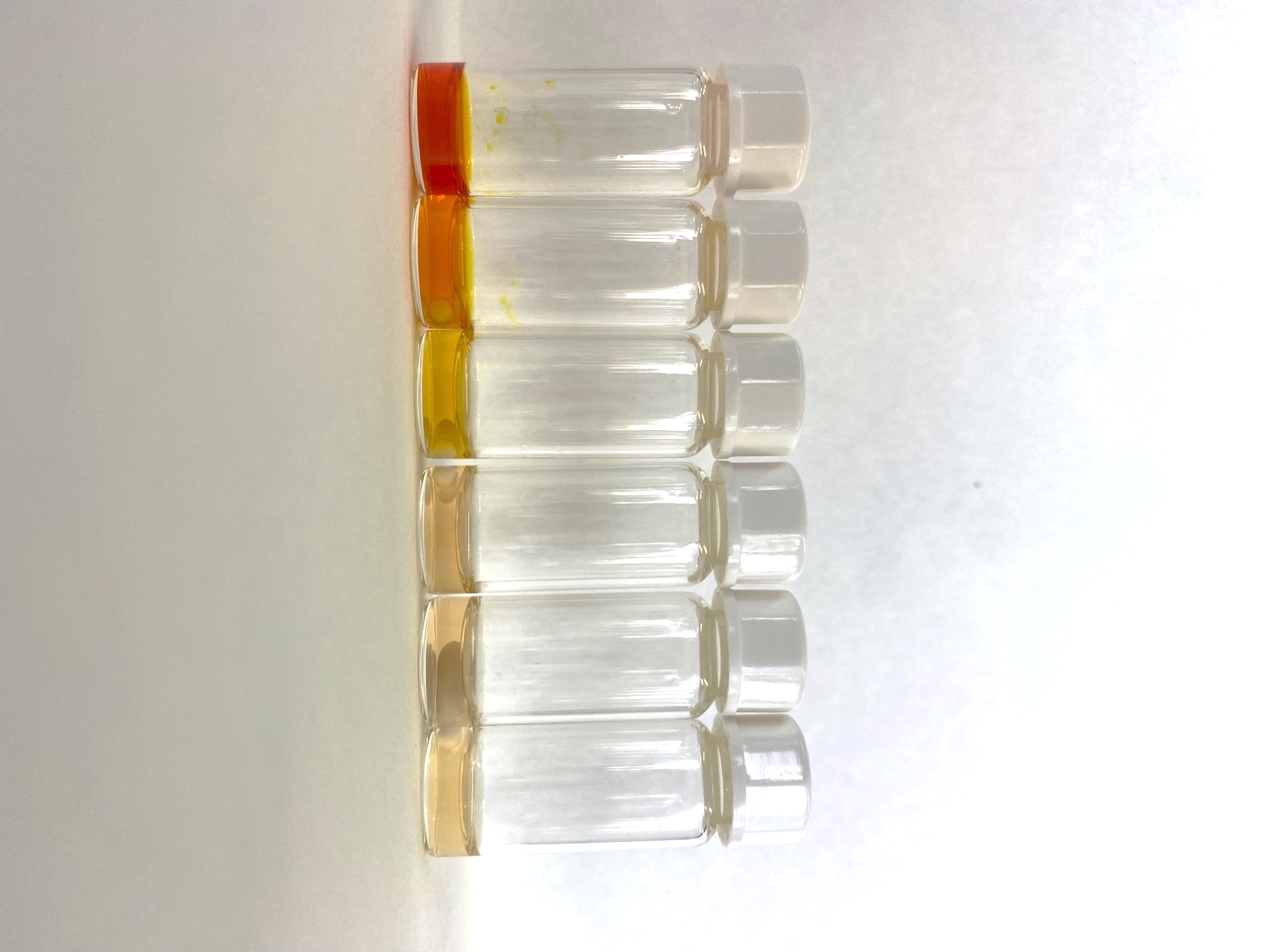


**Figure S7.** Naked-eye changes in the reduction of Compound 2 (10 mM in EtOH) with the treatment of increasing concentrations of hydrazine monohydrate (from left to right, 0-75 equiv.).


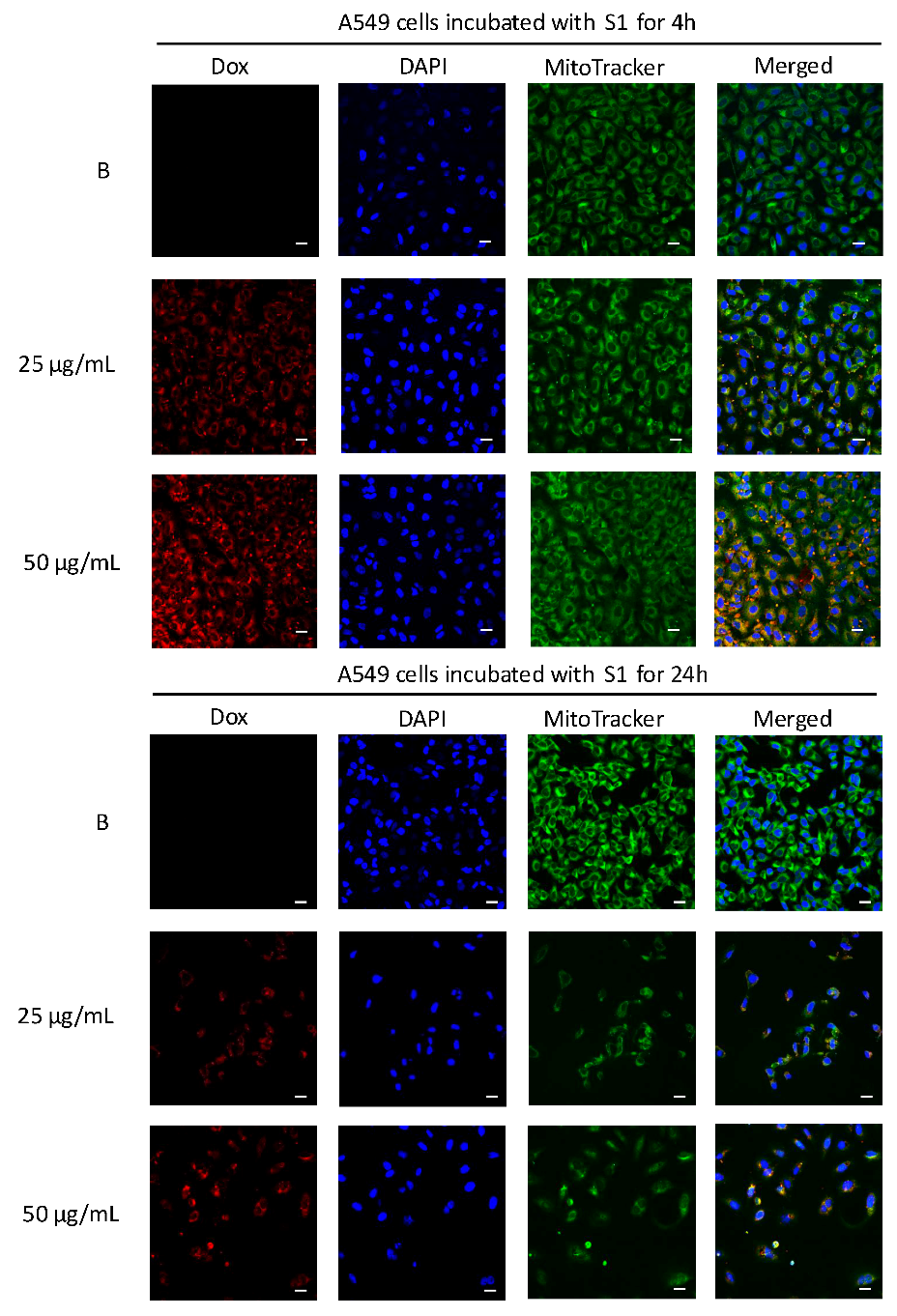


**Figure S8.** Confocal microscope images of A549 untreated cells (B) and A549 cells after incubation with different concentrations of **S1** for 4 and 24 h respectively. Cell nuclei were stained with DAPI and mitochondria with MitoTracker Green. Representative image from three independent experiments. Scale bar 20 μm.


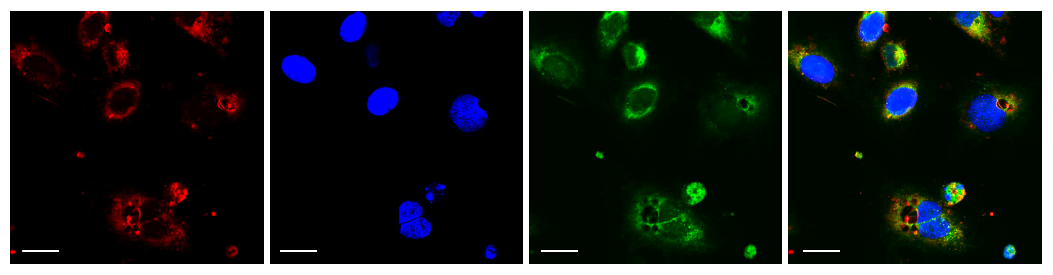


**Figure S9.** 2.2X zoom of the 24 h 50 μg/mL treatment confocal image in Figure S8. Scale bar 20 μm.


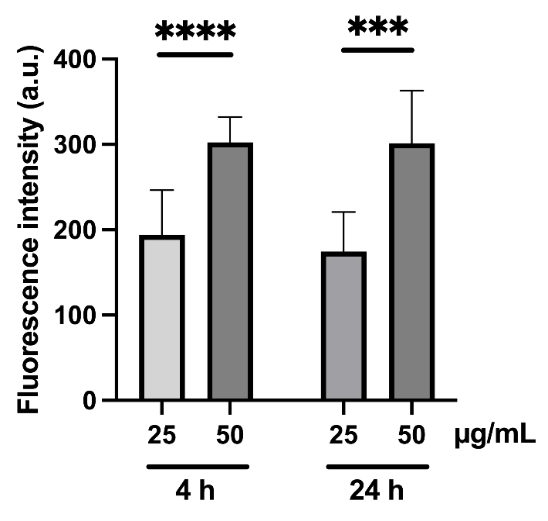


**Figure S10.** Quantitative analysis of Dox fluorescence intensity per field after confocal imaging in A549 cells incubated for 4 and 24h with S1 (25 – 50 μg/mL). Results are expressed as mean ± SD (n = 10).****p<0.0001 Student Test.


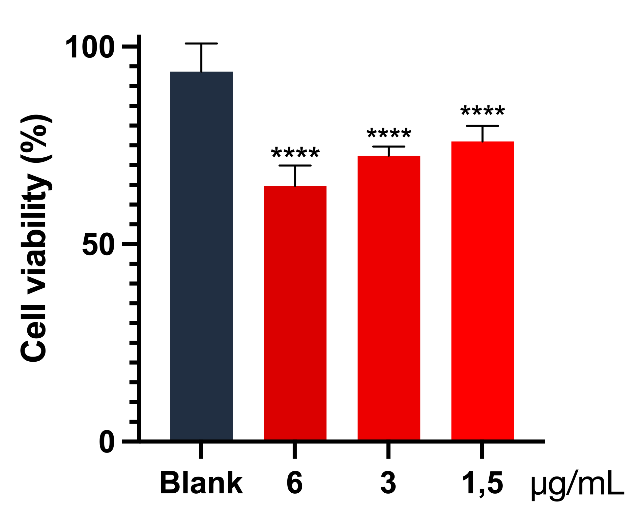


**Figure S11.** Cytotoxicity of free Dox in A549 lung cancer cells after 24h-incubation under hypoxic conditions (2% O_2_). Results are expressed as mean ± SD **(n=3).** ****p< 0.0001 versus Blank untreated cells (B). One-way ANOVA followed by Dunnet’s test.


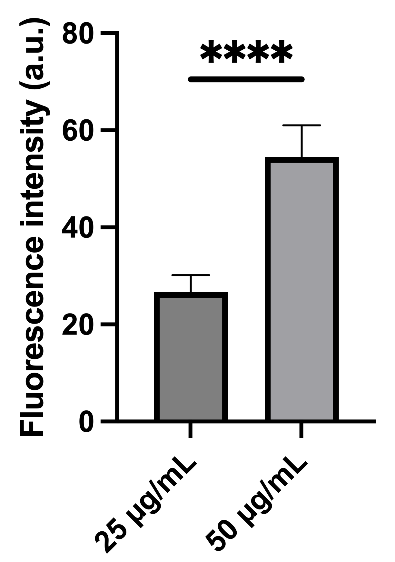


**Figure S12.** Quantitative analysis of Dox fluorescence intensity after confocal imaging in A549 cells incubated for 24h under hypoxia conditions with S1 (25 – 50 μg/mL). Results are expressed as mean ± SD (n = 10).****p<0.0001 Student Test.
